# Aldose Reductase Is Involved in the Development of Murine Diet-Induced Nonalcoholic Steatohepatitis

**DOI:** 10.1371/journal.pone.0073591

**Published:** 2013-09-16

**Authors:** Longxin Qiu, Jianhui Lin, Miao Ying, Weiqiang Chen, Jinmei Yang, Tiantian Deng, Jinfeng Chen, Duanyu Shi, James Y. Yang

**Affiliations:** 1 School of Life Sciences, Longyan University, Longyan, China; 2 Fujian Key Laboratory of Preventive Veterinary Medicine and Biotechnology (Longyan University), Longyan, China; 3 State Key Laboratory of Cellular Stress Biology and School of Life Sciences, Xiamen University, Xiang’an, Xiamen, China; Juntendo University School of Medicine, Japan

## Abstract

Hepatic aldose reductase (AR) expression is known to be induced in liver diseases, including hepatitis and hepatocellular carcinoma. However, the role of AR in the development of these diseases remains unclear. We performed this current study to determine whether and how AR might be involved in the development of diet-induced nonalcoholic steatohepatitis. Our results showed that the level of AR protein expression was significantly higher in db/db mice fed the methionine-choline-deficient (MCD) diet than in mice fed the control diet. In parallel with the elevation in AR, steatohepatitis was observed in MCD diet-fed mice, and this diet-induced steatohepatitis was significantly attenuated by lentiviral-mediated knock-down of the AR gene. This suppressive effect of AR knock-down was associated with repressed levels of serum alanine aminotransferase and hepatic lipoperoxides, reduced mRNA and protein expression of hepatic cytochrome P450 2E1 (CYP2E1), and decreased mRNA expression of pro-inflammatory tumor necrosis factor-α (TNF-α) and interleukin-6 (IL-6). Moreover, AR-induced elevations on the level of CYP2E1 expression, reactive oxygen species, mRNA expression of TNF-α and IL-6 were confirmed in AML12 hepatocytes. Further, lentiviral-mediated knock-down of AR ameliorated MCD diet-induced collagen deposition in the livers of db/db mice. With the improvement in liver fibrosis, the mRNA levels of tissue inhibitor of metalloproteinase-1 (TIMP-1) and matrix metalloproteinase-2 (MMP-2), two genes involved in hepatic fibrogenesis, were found to be significantly suppressed, while TIMP-2 and MMP-13 were unaffected. Together these data indicate that inhibition of AR alleviates the MCD diet-induced liver inflammation and fibrosis in db/db mice, probably through dampening CYP2E1 mediated-oxidative stress and ameliorating the expression of pro-inflammatory cytokines.

## Introduction

The term “nonalcoholic steatohepatitis” (NASH) was first used by Ludwig *et al*. [1] in 1980 to describe the pathological and clinical features of nonalcoholic disease of the liver associated with pathological features most commonly seen in alcoholic liver disease itself. Features of NASH on liver biopsy include steatosis, inflammation, liver cell injury, and varying degrees of fibrosis. It is a more advanced form of nonalcoholic fatty liver disease (NAFLD) and is becoming a major public health problem, but its underlying cause remains unclear. However, there are possible candidates, including insulin resistance [2], [3], toxic inflammatory cytokines [4], and oxidative stress [3], [5] inside liver cells.

Aldose reductase (AR, AKR1B1, EC1.1.1.21) catalyzes the rate-limiting reduction of glucose to sorbitol with the aid of co-factor NADPH [6]. The role of AR in the development of diabetic complications is well-established. Moreover, AR expression has been found to be induced in some tissues in some disease conditions other than diabetes. Interestingly, AR is induced in diseased liver. O’Connor et al. reported that AR was detectable in the livers of two human subjects with alcoholic liver disease, while it was undetectable in healthy humans [7]. Similarly, neither AR mRNA nor protein is detectable in the livers of Long-Evans cinnamon rats prior to the development of hepatitis or in the livers of a closely related rat strain that does not develop hepatitis, while expression of AR in the livers of the cinnamon rats showed a temporal relationship with the onset of liver injury [8]. Further, Brown et al. reported that AR was induced in human livers obtained from patients undergoing liver transplantation for fulminant (acute) liver failure or for end-stage liver disease from cirrhosis due to various chronic liver diseases, including chronic hepatitis B and C, alcoholic liver disease, primary biliary cirrhosis, autoimmune hepatitis, and hepatocellular carcinoma [9].

These findings raise the question of whether AR is involved in the development of hepatitis and fibrosis. In this study, we examined the role of AR in the development of nutrition-induced murine NASH by lentiviral-mediated knock-down of the AR gene in db/db mice fed a methionine-choline-deficient (MCD) diet and investigated the mechanism whereby AR participates in the development of NASH.

## Materials and Methods

### Lentivirus shRNA Construct

Recombinant lentiviral vectors expressing a small hairpin RNA (shRNA) against mouse AR (pLV-shAR) and its control (pLV-shNC) were constructed as described previously [10]. Lentivirus preparations were performed by cotransfecting the lentiviral constructs with packaging vectors into 293 T cells using Lipofectamine 2000 (Invitrogen). Virus-containing supernatants were collected 48 h after infection. Viruses were recovered by ultracentrifugation (110,000×*g*, 1.5 h, 4°C) and resuspended in PBS. Titers were determined by infecting 293 T cells with serial dilutions of concentrated lentiviral preparations.

### Animal Experiments

All experiments were conducted according to protocols and guidelines approved by Xiamen University Institutional Animal Care and Use Committee. The db/m (BKS.Cg-m^+/+^Lepr^db^/J) mice were obtained from the Jackson Laboratory (Bar Harbor, Maine) and bred to obtain eight week old female homozygous db/db (Lepr ^db/db^) mice for this study. db/db mice are mutant mice that carry naturally-occuring genetic mutations in the leptin receptor gene. As the consequence of the loss of leptin receptor activity, db/db mice normally become obese and diabetic 6–8 weeks after birth. db/db mice fed the MCD diet was used as a useful small animal model of progressive NAFLD. Feeding mice with a lipogenic MCD diet was previously shown to be capable of inducing liver injury similar to human NASH. MCD-fed db/db mice are therefore good models for NASH [11]. All animals were maintained on standard laboratory chow under a 12/12-h light/dark schedule. db/db mice were randomly grouped (6 mice/group) and injected with either pLV-shAR or pLV-shNC and then maintained on the MCD diet (MP Biomedicals, Aurora, OH) or a control diet for 8 weeks before being sacrificed. *In vivo* transduction of lentiviruses was achieved through tail vein injections of 0.1 mL of concentrated viral suspension with a viral titer of 1.0×10^9^ IFU/mL lentiviral particles in PBS; injections were conducted once every 4 weeks.

### Analyses of mRNA Expression by Quantitative Real-time PCR

Total RNA was isolated from tissues or cells using the Trizol reagent (Invitrogen) according to the manufacturer’s protocol. Complementary DNA (cDNA) was synthesized from hepatic mRNA using RevertAid First Strand cDNA Synthesis kits (Fermentas). Hepatic cytochrome P450 2E1 (CYP2E1), tumor necrosis factor-α (TNF-α), interleukin-6 (IL-6), transforming growth factor-β1 (TGF-β1), tissue inhibitor of metalloproteinase-1 (TIMP-1), TIMP-2, matrix metalloproteinase-2 (MMP-2), MMP-13, and collagen I mRNA were analyzed as described previously [12], [13] with the specific primers listed in Table 1. Quantitative real-time polymerase chain reactions were performed using the FastStart Universal SYBR Green Master (Rox) (Roche Applied Science). Each Ct value was calibrated with that for 18S rRNA.

**Table 1:** Primer sequences used for amplification of mRNA by real-time PCR.

Gene	Sequences	Accession No.
CYP2E1	F 5′ – AGTGTTCACACTGCACCTGG –3′	BC042693
	R 5′ – CCTGGAACACAGGAATGTCC –3′	
TNF-α	F 5′ – CGTGCTCCTCACCCACAC –3′	NM013693
	R 5′ – GGGTTCATACCAGGGTTTGA –3′	
IL-6	F 5′ – ACAACCACGGCCTTCCCTACTT –3′	NM031168
	R 5′ – GTGTAATTAAGCCTCCGACT –3′	
TGF-β1	F 5′ – CAACTTCTGTCTGGGACCCT –3′	NM011577
	R 5′ – TAGTAGACGATGGGCAGTGG –3′	
Collagen I	F 5′ – ATGTTCAGCTTTGTGGACCTC –3′	BC003198
	R 5′ – TCCCTCGACTCCTACATCTTC –3′	
TIMP-1	F 5′ – CATGGAAAGCCTCTGTGGATATG –3′	BC008107
	R 5′ – GATGTGCAAATTTCCGTTCCTT –3′	
TIMP-2	F 5′ – ATGGTTCTTGCGCGTGGTA –3′	X62622 S37984
	R 5′ – GCTTTTCAATTGGCCACAGG –3′	
MMP-2	F 5′ – GATACCCTCAAGAAGATGCAGAAGTT –3′	M84324
	R 5′ – ACATCTTGGCTTCCGCATG –3′	
MMP13	F 5′ – ACTTAACTTACAGGATTGTGAACTATACTCCT –3′	X66473 S48798
	R 5′ – TGTCAGCAGTGCCATCATAGATT –3′	
18S rRNA	F 5′ – CGACGACCCATTCGAACGTCT –3′	NR003278
	R 5′ – CTCTCCGGAATCGAACCCTGA –3′	

### Western Blot Analyses

Tissues were homogenized in ice-cold RIPA buffer. Each protein sample (40 µg) was loaded and separated on a 12% SDS-polyacrylamide gel and transferred to polyvinylidene difluoride (PVDF) membranes (Millipore). Blotted membranes were then incubated with anti-AR (Santa Cruz Biotechnology; 1∶500) or anti-CYP2E1 (Abcam; 1∶1000) in TBS - 0.1% Tween-20 with 5% non-fat milk at 4°C overnight. After several washes, the membranes were incubated with horseradish peroxidase-conjugated anti-goat IgG or anti-rabbit IgG (Sigma; 1∶2000) in TBS - 0.1% Tween-20 with 5% non-fat milk. Detection was achieved using the Supersignal chemiluminescent substrate kit (Pierce).

### Histological Examination

Formalin-fixed liver tissue was processed and 5-µm-thick paraffin sections were stained with hematoxylin and eosin (H&E) and Masson’s trichrome for histological analyses. A hepatopathologist who was blinded to the experimental conditions examined all sections for steatosis, inflammation, and fibrosis [14]. Hepatic necroinflammation were graded on H&E-stained sections and was given a score from 0 to 3, as follows: 0: no inflammatory foci, 1: mild, 2: moderate, and 3: severe. The degree of fibrosis was assessed on Masson’s trichrome-stained sections by digital morphometry, as described previously [11].

### Tissue and Serum Biochemical Measurements

Serum alanine aminotransferase (ALT) was measured using spectrophotometric assay kits (Nanjin Jiancheng, China). Total lipoperoxides were measured as thiobarbituric acid reactive substances (TBARS) in 100 µL of liver homogenate using commercial kits (Beyotime, China). TBARS were quantified using malondialdehyde (MDA) as a standard.

### Cell Culture

AML12 mouse hepatocytes cells were obtained from ATCC and cultured as instructed. AML12 cells were plated on 6-well plates at the cell density of approximately 1.0×10^6^/well. On the next day, cells were transfected with pFLAG-mAR, a plasmid constructed as described previously [15] or pFLAG-CMV2 (Sigma) using Lipofectamine 2000 reagent (Invitrogen) according to the manufacturer’s protocol. 36 hours later, cells were collected for mRNA expression analyses.

### Detection of Reactive Oxygen Species (ROS) Generation in AML12 Cells

36 hours after pFLAG-mAR or pFLAG-CMV2 transfection as described above, cells were washed and resuspended in PBS buffer and equilibrated with 10 µM DCF-DA for 1 h at 37°C. Thereafter, 10^6^ cells/200 µL/well were loaded in black 96-well micro plate and the DCF fluorescence was measured in a fluorescence microplate reader (Varioskan Flash, Thermo Scientific) using excitation and emission wavelengths of 485 and 530 nm, respectively.

### Statistical Analyses

All data were processed and analyzed using the GraphPad software (Prism 5.0) and are expressed as means ± SEM. Student’s *t*-test was used for pair-wise comparisons and one-way ANOVA with Bonferroni’s Multiple Comparison Test was used for multi-group analyses. Probability values less than 0.05 (*) were considered to indicate statistical significance; those less than 0.01 (**), more so.

## Results

### Hepatic AR was Induced in MCD Diet-fed db/db Mice and Lentiviral-mediated Knock-down of AR Gene Attenuated Diet-induced Steatohepatitis Significantly

**Figure 1:**
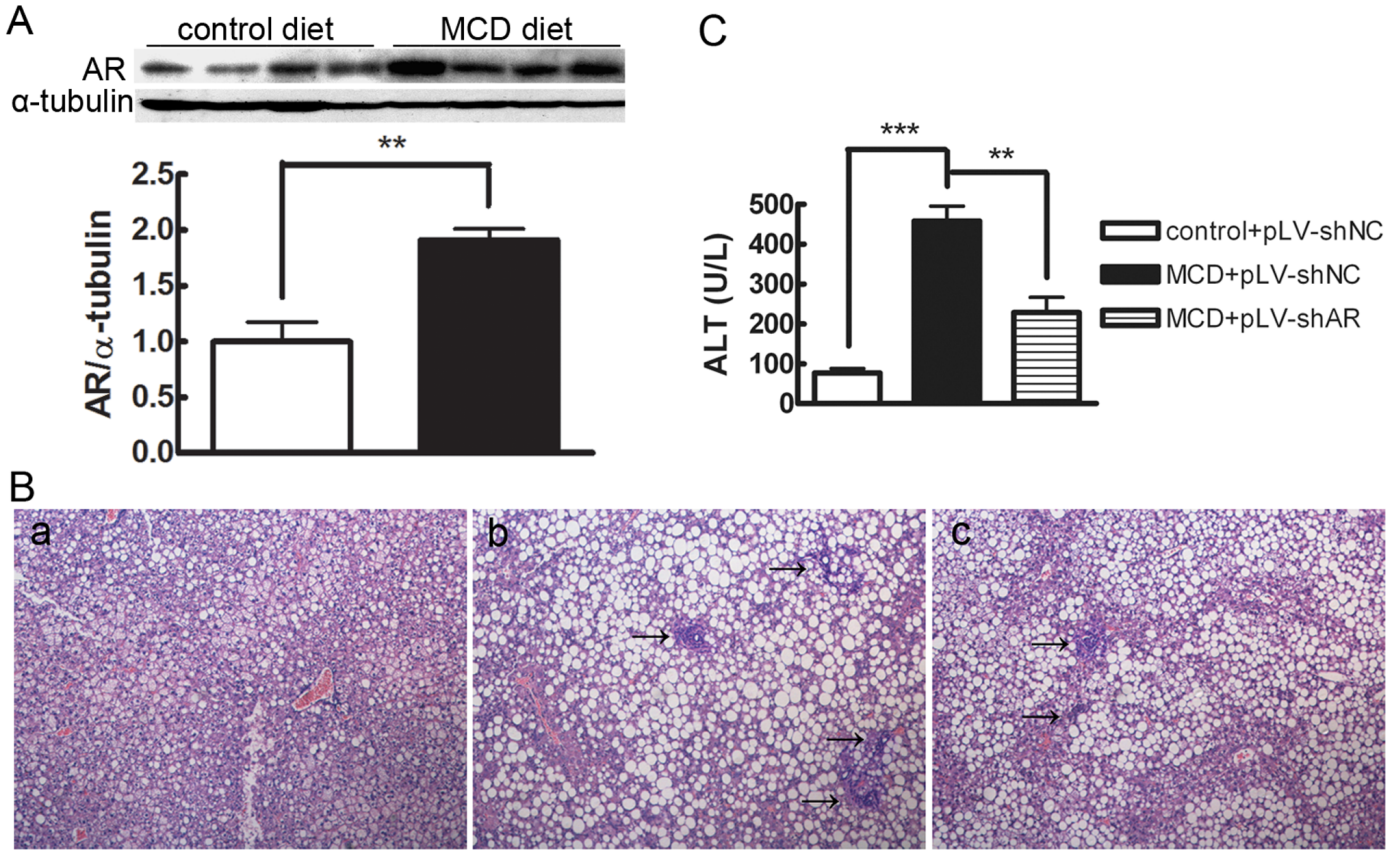
AR is involved in the development of MCD diet-induced steatohepatitis in db/db mice. A. Representative Western blot shows the induction of hepatic AR protein expression in MCD diet-fed mice. Average densitometric analyses of AR were calculated as fold increases over the control diet (*n = *4); values are expressed as the mean ± SEM. **, P<0.01. B. Hematoxylin and eosin–stained liver sections from mice fed: (a) Control diet+pLV-shNC. (b) MCD diet+pLV-shNC. (c) MCD diet+pLV-shAR. Arrows point to foci of necroinflammation. Slides are representative of four separate experiments (original magnification, ×100). C. Effect of MCD diet and lentiviral-mediated knock-down of AR on serum ALT levels in db/db mice. Data are means ± SEM of six mice in each group. ***, P<0.001; **, P<0.01.

Previous studies showed that feeding db/db mice MCD diets induce NASH and liver fibrosis within 4–8 weeks [11], [14]. To investigate whether AR was involved in the development of MCD diet-induced steatohepatitis, we first detected protein expression levels of hepatic AR in db/db mice fed the MCD diet. As shown in Figure 1A, hepatic AR protein expression in db/db mice fed the MCD diet for 4 weeks was ∼91% higher (P<0.01) than that in mice fed the control diet. A similar elevation of hepatic AR was also observed after feeding MCD diet for 8 weeks (data not shown). To further determine the role of AR in the development of diet-induced steatohepatitis, we knocked down the AR gene by *in vivo* transduction of a lentiviral vector carrying shRNA against the mouse AR gene through tail vein injections. As shown in Figure 1B and Table 2, examination of H&E-stained sections demonstrated marked lobular inflammation in db/db mice fed the MCD diet for 8 weeks while db/db mice fed the control diet did not exhibit significant histological inflammation. After knocking down the AR gene (the efficiency of knock-down was 66.7% as determined by Western blotting; data not shown), lobular inflammation in db/db mice fed the MCD diet was attenuated significantly (P<0.01). However, AR knock-down did not improve the steatosis in db/db mice fed the MCD diet. Consistent with the histological findings, AR knock-down resulted in a significant decrease in serum ALT levels in db/db mice fed the MCD diet (P<0.01; Fig. 1C). In parallel with the AR knockdown db/db mice, in C57BL/6 mice deficient in AR, we found that genetic ablation of AR gene significantly improved MCD diet induced-steatohepatitis in non-obesity and non-diabetic mice (Fig. S1). Together, these data indicate that AR is involved in the development of MCD diet-induced steatohepatitis.

**Table 2:** Effect of AR knock-down on scores for hepatic necroinflammation and fibrosis.

Measurements	Controls+pLV-shNC	MCD+pLV-shNC	MCD+pLV-shAR
necroinflammation	0.00±0.00	3.00±0.77	0.67±0.33*
Central vein fibrosis	0.13±0.03	0.62±0.10	0.28±0.06*
Portal tract fibrosis	0.03±0.01	0.59±0.09	0.22±0.05*

The severity of hepatic necroinflammation and fibrosis were scored as described in the Materials and Methods. Values are means ± (*n* = 4/group).

* P<0.05 compared with MCD+pLV-shNC.

### AR was Involved in the Control of Hepatic Oxidative Stress Status in db/db Mice Fed the MCD Diet

**Figure 2:**
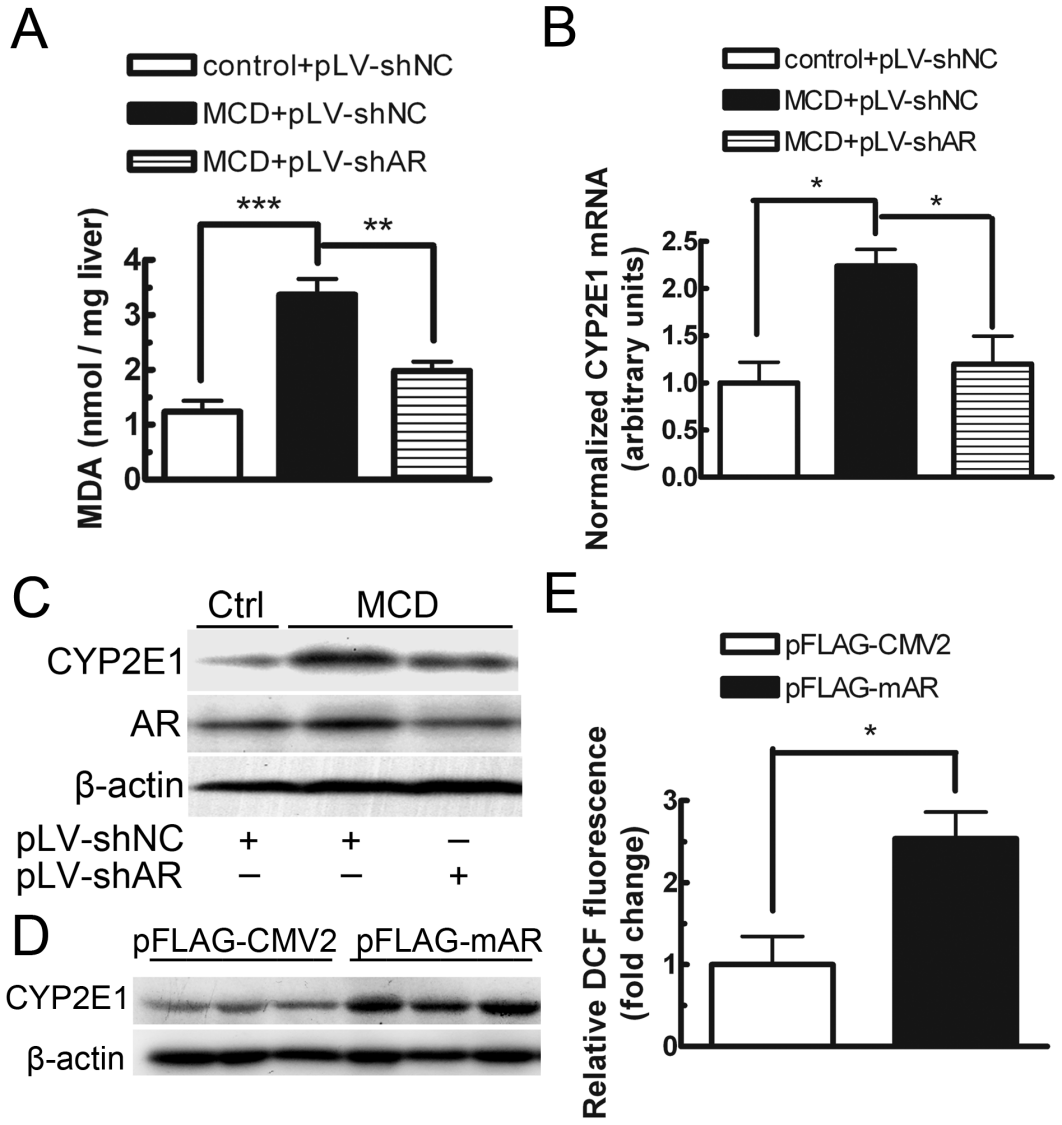
Effect of lentiviral-mediated knock-down of AR on hepatic lipoperoxide content, CYP2E1 expression in db/db mice and over-expression of AR on CYP2E1 expression and ROS levels in AML12 cells. Hepatic lipoperoxide content (A), CYP2E1 mRNA (B) and protein (C) expression were determined in mice fed the control diet, MCD diet, or MCD diet treated with lentiviruses carrying shRNA against AR for 8 weeks (*n = *4). CYP2E1 protein expression (D) and ROS levels (E) were assayed in AML12 cells transfected with pFLAG-mAR for 36 hours (*n = *3). Values are expressed as the mean ± SEM. ***, P<0.001; **, P<0.01; *, P<0.05.

To clarify the mechanism of AR’s involvement in the development of diet-induced steatohepatitis, we first investigated the effect of AR knock-down on hepatic lipoperoxide production. As shown in Figure 2A, intake of the MCD diet resulted in a prominent increase in hepatic TBARS levels, compared with that of the control diet, and the increase was attenuated significantly by treating the MCD diet-fed mice with lentiviruses containing the AR-shRNA expression cassette (P<0.05). Meanwhile, the mRNA and protein expression of CYP2E1, a major mediator of lipid peroxidation [16], [17], were induced by the MCD diet, and AR knock-down significantly prevented the induction of CYP2E1 (Fig. 2B, C). In addition, to further determine whether the induced oxidative stress is the consequence of AR elevation, we over-expressed AR in mouse AML12 hepatocytes by transfecting an AR express vector, pFLAG-mAR into the cells. As shown in Fig. 2D&E, over-expression of AR resulted in an induction of CYP2E1 protein and a 2.5-fold increase in ROS levels (P<0.05). Together, these data indicate that overactivation of AR might promote oxidative stress in the liver in mice with MCD-diet induced NASH, and the increased hepatic oxidative stress is at least in part attributed to the AR-mediated induction of CYP2E1.

### AR Modulated the mRNA Expression of Some Inflammatory Mediators in the Liver of MCD Diet-fed Mice and in Hepatocytes

**Figure 3:**
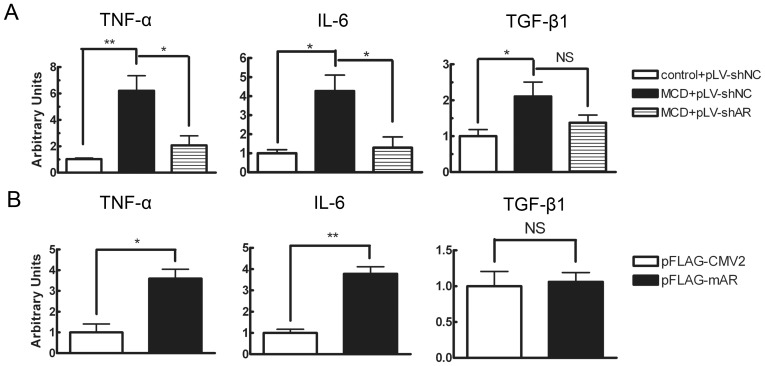
Effect of AR on hepatic mRNA expression of proinflammatory cytokines. Total RNA was isolated and analyzed for TNF-α, IL-6 and TGF-β1 in db/db mice (A) fed the control or MCD diet and transduced with pLV-shNC or pLV-shAR for 8 weeks (*n = *4) and in AML12 cells (B) transfected with pFLAG-CMV2 or pFLAG-mAR for 36 hours (*n = *3). Values are expressed as the mean ± SEM. **, P<0.01; *, P<0.05.

To evaluate the effect of AR elevation on the development of inflammation, we investigated the expression levels of some proinflammatory cytokines including TNF-α, IL-6 and TGF-β1. Mice fed the MCD diet had a marked elevation of hepatic mRNA expression of TNF-α, IL-6 and TGF-β1 as compared with mice fed the control diet (Fig. 3A). However, the diet-induced TNF-α and IL-6 mRNA elevation were significantly attenuated, by 66.7% (P<0.05) and 69.8% (P<0.05), respectively, in db/db mice treated with lentiviruses carrying shRNA for AR. In comparison with TNF-α and IL-6, the effect of AR knock-down on TGF-β1 was less significant. Consistent with the *in vivo* finding, AR over-expression in AML12 cells resulted in a significant increase in the mRNA expression levels of TNF-α (P<0.05) and IL-6 (P<0.01) but not TGF-β1 (Fig. 3B). Taken together, these data suggest that hepatic AR elevation in db/db mice fed the MCD diet might directly affect the production of pro-inflammatory cytokines TNF-α and IL-6 in the liver.

### AR Knock-down Reduced the Development of Diet-induced Liver Fibrosis

**Figure 4:**
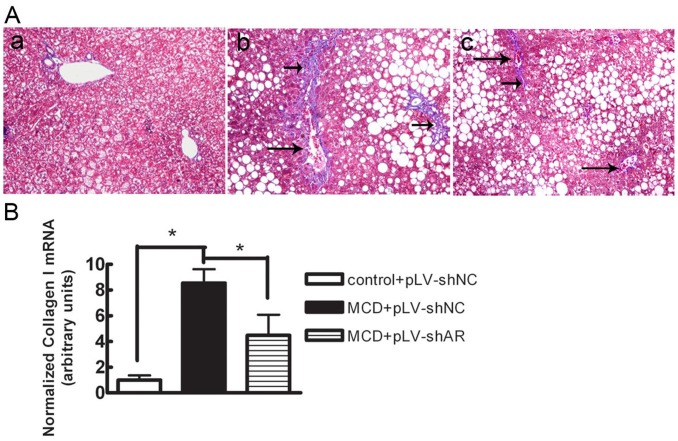
Effect of lentiviral-mediated knock-down of AR on MCD diet–induced collagen deposition in db/db mice. A. Masson’s trichrome–stained liver sections from mice fed (a) the control diet+pLV-shNC, (b) the MCD diet+pLV-shNC, and (c) the MCD diet+pLV-shAR. Long arrows point to perivenular and small arrows to pericellular fibrosis. Slides are representative of four separate experiments (original magnification, ×200). B. Total RNA was isolated and analyzed for collagen type I mRNA expression. Values are expressed as the mean ± SEM. *, P<0.05.

Trichrome staining and collagen I mRNA expression were assessed as indices of liver fibrosis. As shown in Figure 4A and Table 2, mice on the control diet showed insignificant levels of hepatic fibrosis. In contrast, MCD diet-fed mice showed a 4.8-fold increase in central/pericentral vein fibrosis and a 19.7-fold increase in portal/periportal fibrosis when compared with control diet-fed mice. pLV-shAR- transduced mice showed a significant decrease in central/pericentral vein fibrosis, by 54.8% (P<0.05), and in portal/periportal fibrosis, by 62.7% (P<0.05), compared with pLV-shNC-transduced mice. In parallel with the changes in histological fibrosis, the MCD diet induced an increase of 8.5-fold in hepatic collagen I mRNA expression in db/db mice, and AR knock-down attenuated the diet-induced elevation of collagen I mRNA, by 47.5% (P<0.05; Fig. 4B). Together, these data indicate that the lentiviral-mediated knock-down of AR reduced the development of MCD diet-induced liver fibrosis.

### AR Knock-down Affected the Hepatic Expression of Genes Involved in the Development of Fibrosis

**Figure 5:**
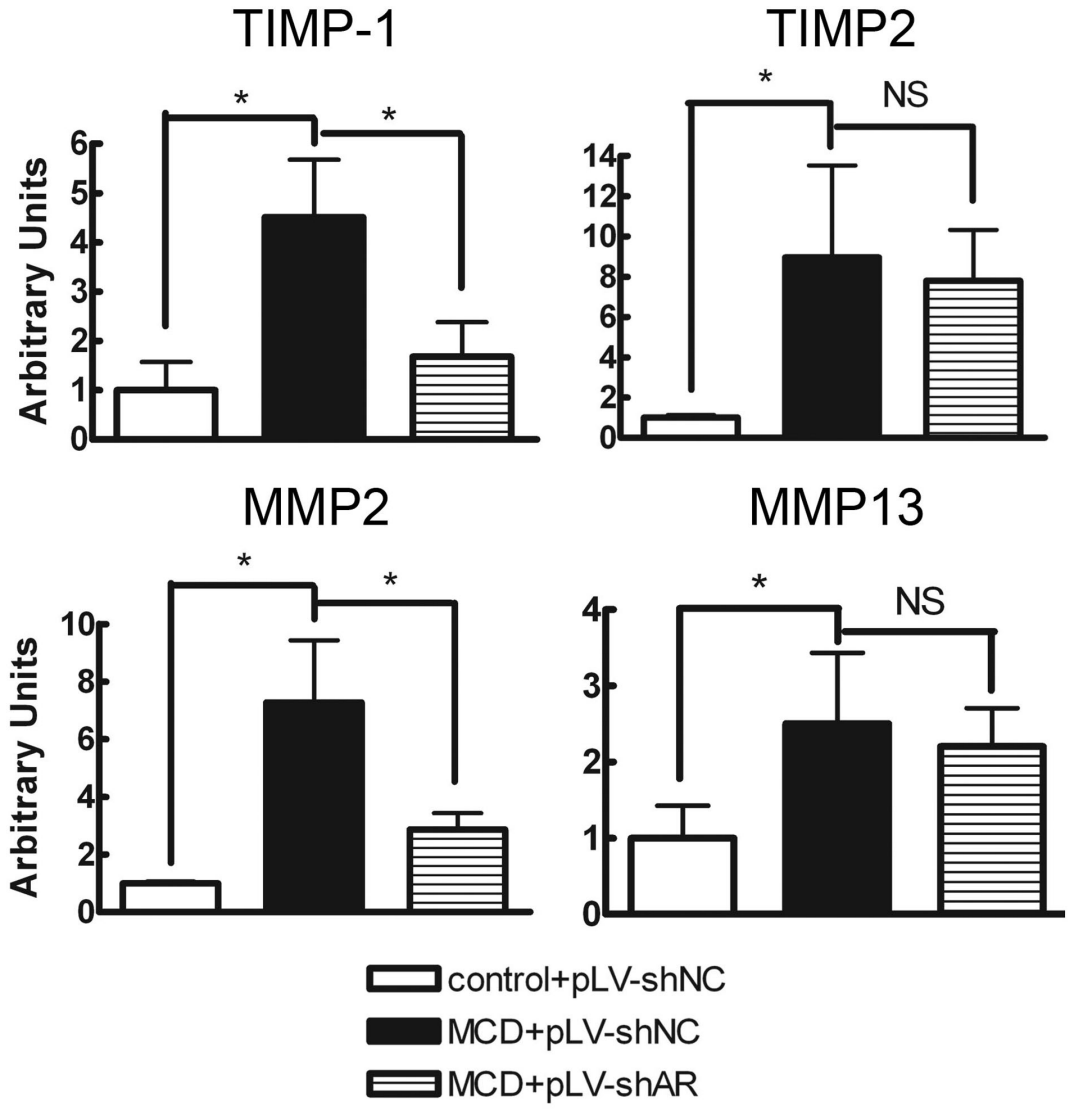
Effect of lentiviral-mediated knock-down of AR on hepatic mRNA levels of genes involved in liver fibrogenesis. Hepatic mRNA levels were assessed using reverse transcription-real time PCR, standardized against an internal control (18S rRNA) and are expressed as fold differences compared with values obtained in mice fed the control diet (*n = *4). Values are expressed as the mean ± SEM. *, P<0.05.

To clarify the mechanism through which AR knock-down reduced liver fibrosis, we examined molecular pathways involved in matrix remodelling and degradation to determine whether AR knock-down improved matrix remodelling or stimulated matrix degradation at the transcriptional level. As shown in Figure 5, in control diet-fed mice, there was only a low level of hepatic expression of genes for inhibitors of fibrosis reversal (TIMP-1, TIMP-2) and those involved in matrix degradation (MMP-2, MMP-13). The MCD diet increased the expression of transcripts for these proteins. AR knock-down significantly reduced mRNA expression of TIMP-1, by 62.7% (P<0.05), while TIMP-2 mRNA was unaffected. MMP-2 expression was not increased, but was significantly reduced, by 60.5% (P<0.05), while MMP-13 mRNA levels were unaffected.

## Discussion

AR induction has been observed in some liver diseases conditions, including alcoholic liver disease, chronic hepatitis B and C, and hepatocellular carcinoma in humans and in hereditary hepatitis in rats [7]–[9]. However, the role of AR in the development of these liver diseases has remained unclear. In this study, we first demonstrated that hepatic AR protein was also induced in the MCD-diet induced steatohepatitis condition in db/db mice. Then, we conducted *in vivo* transduction of lentiviruses carrying shRNA for AR in the MCD diet-fed db/db mice to examine the role of AR in the development of diet-induced NASH. We demonstrated that the lentiviral-mediated knock-down of AR improved the development of steatohepatitis and liver fibrosis. Our results clearly indicate that AR is involved in the development of NASH.

In addition to glucose, AR can catalyze the reduction of a variety of aldehydes and carbonyls, such as 4-hydroxy-2-nonenal (4HNE) [18]. 4HNE is a cytotoxic byproduct of lipid peroxidation that is believed to participate in the pathogenesis of a variety of pathological conditions [19]. Thus, AR has been postulated to serve a cytoprotective function by rapidly detoxifying aldehydes. Indeed, *in vitro* studies have shown that AR expression is induced by 4HNE in rat vascular smooth muscle cells and inhibition of AR sensitizes cells to 4HNE cytotoxicity [20]. Further, an *in vivo* study showed that inhibition of AR was associated with increased numbers of apoptotic cells as well as 4HNE content in the inflamed arterial wall in a murine model of giant cell arteritis [21]. However, AR inhibitors have also been reported to exert beneficial effects on injuries in a variety of other rodent models, including allergic airway inflammation, ischemic myocardial injury, arterial balloon injury, and uveitis [22]–[25]. In this study, we demonstrated that AR knock-down attenuated lipid peroxidation in a murine model of steatohepatitis. Our data suggest that AR elevation in mice with steatohepatitis exacerbated the oxidative stress status and, in turn, affected the progression of steatohepatitis. The mechanisms by which AR inhibition or knock-down exert beneficial effects in these injuries remain to be determined, because AR also possesses the capacity to rapidly detoxify aldehydes. However, we demonstrated that AR knock-down prevented the induction of CYP2E1 in MCD diet-fed db/db mice. CYP2E1 plays an important role in the pathogenesis of liver tissue injury [16], [17]. There is accumulating evidence that upregulation of CYP2E1 may initiate lipid peroxidation by the production of reactive oxygen species [26]. Thus, this capacity for preventing the induction of hepatic CYP2E1 in mice with steatohepatitis might account, partly, for the mechanism by which AR knock-down attenuates lipid peroxidation and ameliorates steatohepatitis.

The molecular mechanisms leading from liver steatosis to NASH still remain unclear. Among the candidates, the contribution of inflammatory cytokines, such as TNF-α and/or IL-6, seems obvious. Liver TNF-α and TNF receptor 1 (TNFR1) mRNA [27] are increased in patients with NASH. Additionally, liver steatosis and fibrosis were reported to be attenuated in TNFR1/TNFR2-deficient mice fed the MCD diet [28]. However, some studies have lead to conflicting conclusions. For example, a deficiency in TNF receptors did not prevent the elevation of serum ALT in ob/ob mice [29] or in high-fat diet overfed mice [30]. Regarding IL-6, a pleiotropic cytokine that regulates inflammatory responses and that is another putative mediator of steatohepatitis, its precise role in NASH is also uncertain. Treatment of mice with IL-6 ameliorated steatosis in different models of fatty liver, including ob/ob mice and ethanol-fed mice [31], [32]. However, IL-6 expression is increased in the liver of patients with NASH and correlates with disease severity [33]. Moreover, genetic deletion of IL-6 markedly attenuated hepatic inflammation in MCD diet-induced NASH [34]. Thus, the role of inflammatory cytokines in the development of NASH has not yet been fully explained. It seems more likely that inflammatory cytokines are not primary mediators of NASH but influence its development. In the current study, we demonstrated that the levels of TNF-α and IL-6 correlated with the expression level of AR in MCD diet-fed mice. Thus, our data suggest that AR might mediate the production of TNF-α and IL-6 to influence the development of MCD diet-induced liver inflammation.

In liver tissue, MMPs and their specific inhibitors, the TIMPs, play a pivotal role in both fibrogenesis and fibrolysis. Consistent with a previous study in mice fed the MCD diet [12], expression levels of MMP-2, MMP-13, TIMP-1, and TIMP-2 were increased in mice with MCD diet-induced hepatic fibrosis in this study. Knocking down AR led to down-regulation of TIMP-1 and MMP-2 expression, while TIMP2 and MMP-13 expression was maintained. The imbalance between MMPs and their inhibitors thus tends to affect the development of fibrosis. Of note, there are species differences in the cellular localization of AR in the liver. Immunohistochemical studies failed to identify AR in either quiescent or activated human hepatic stellate cells (HSCs) [9], whereas AR was identified in rat HSCs [35], an important nonparenchymal cell type involved in the production of MMPs and TIMPs. Thus, whether AR directly affects the expression of TIMP1 and MMP2 to affect fibrosis remains to be determined. It seems more likely that AR knock-down reverses fibrosis by reducing profibrogenic stimuli, such as lipoperoxides and some cytokines. Lipoperoxides are regulators of collagen I gene expression [36] and TNF-α and IL-6 stimulate HSC activity and increase the production of extracellular matrix components [37], [38]. Thus, the beneficial effects of AR knock-down in reducing lipoperoxides and proinflammatory cytokines may contribute, at least in part, to the amelioration of MCD diet-induced liver fibrosis.

## Supporting Information

Figure S1 **Ablation of AR gene improved MCD diet-induced steatohepatitis in C57BL/6 mice.** A. Hematoxylin and eosin–stained liver sections from: (a) AR^+/+^ mice fed control diet. (b) AR^+/+^ mice fed MCD diet. (c) AR^−/−^ mice fed MCD diet. Arrows point to foci of necroinflammation. Slides are representative of four separate experiments (original magnification, ×100). B. Effect of knock-out of AR on serum ALT levels in C57BL/6 mice. Data are means ± SEM of six mice in each group. **, P<0.01.
(TIF)
