# Supplementary figures and images for: Aldose Reductase Is Involved in the Development of Murine Diet-Induced Nonalcoholic Steatohepatitis
Source: PLoS One. 2013 Sep 16;8(9):e73591. doi: 10.1371/journal.pone.0073591 (PMC3774689; doi:10.1371/journal.pone.0073591)

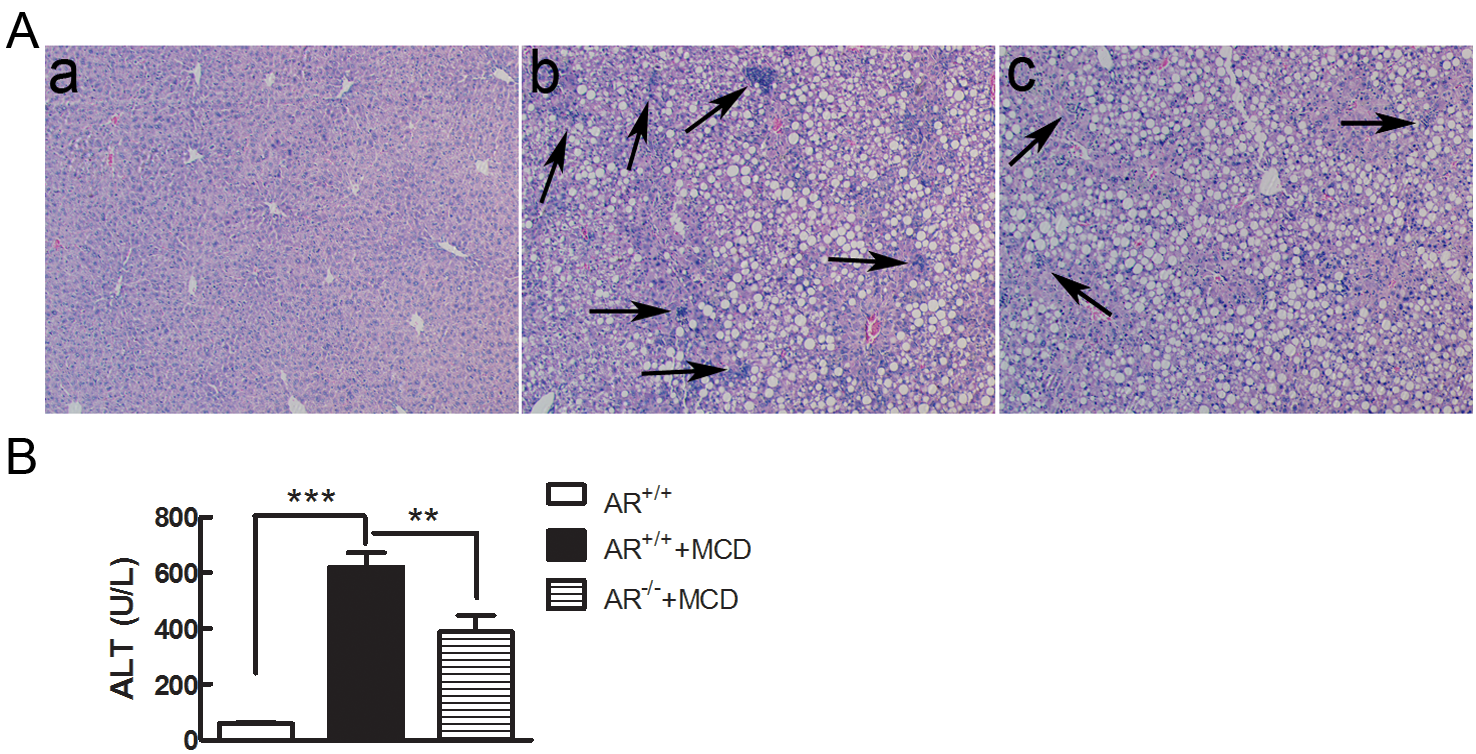

Supplement: Figure S1 — Ablation of AR gene improved MCD diet-induced steatohepatitis in C57BL/6 mice. A. Hematoxylin and eosin–stained liver sections from: (a) AR+/+ mice fed control diet. (b) AR+/+ mice fed MCD diet. (c) AR−/− mice fed MCD diet. Arrows point to foci of necroinflammation. Slides are representative of four separate experiments (original magnification, ×100). B. Effect of knock-out of AR on serum ALT levels in C57BL/6 mice. Data are means ± SEM of six mice in each group. **, P<0.01. (TIF) [file pone.0073591.s001.tif]
